# EEG source imaging concordance with intracranial EEG and epileptologist review in focal epilepsy

**DOI:** 10.1093/braincomms/fcab278

**Published:** 2021-11-19

**Authors:** Benjamin C Cox, Omar A Danoun, Brian Nils Lundstrom, Terrence D Lagerlund, Lily C Wong-Kisiel, Benjamin H Brinkmann

**Affiliations:** 1 Department of Neurology, Mayo Clinic, Rochester, MN 55905, USA; 2 Department of Neurology, Henry Ford Hospital, Detroit, MI 48202, USA

**Keywords:** EEG source imaging, source localization, intracranial EEG, epilepsy surgery

## Abstract

EEG source imaging is becoming widely used for the evaluation of medically refractory focal epilepsy. The validity of EEG source imaging has been established in several studies comparing source imaging to the surgical resection cavity and subsequent seizure freedom. We present a cohort of 87 patients and compare EEG source imaging of both ictal and interictal scalp EEG to the seizure onset zone on intracranial EEG. Concordance of EEG source imaging with intracranial EEG was determined on a sublobar level and was quantified by measuring the distance between the source imaging result and the centroid of the active seizure onset zone electrodes. The EEG source imaging results of a subgroup of 26 patients with high density 76-channel EEG were compared with the localization of three experienced epileptologists. Of 87 patients, 95% had at least one analysis concordant with intracranial EEG and 74% had complete concordance. There was a higher rate of complete concordance in temporal lobe epilepsy compared to extratemporal (89.3 and 62.8%, respectively, *P* = 0.015). Of the total 282 analyses performed on this cohort, higher concordance was also seen in temporal discharges (95%) compared to extratemporal (77%) (*P* = 0.0012), but no difference was seen comparing high-density EEG with standard (32-channel) EEG. Subgroup analysis of ictal waveforms showed greater concordance for ictal spiking, compared with rhythmic activity, paroxysmal fast activity, or obscured onset. Median distances from the dipole and maximum distributed source to a centroid of seizure onset zone electrodes were 30.0 and 32.5 mm, respectively, and the median distances from dipole and maximum distributed source to nearest seizure onset zone electrode were 22.8 and 21.7, respectively. There were significantly shorter distances in ictal spiking. There were shorter distances in patients with Engel Class 1 outcome from surgical resection compared to patients with worse outcomes. For the subgroup of 26 high-density EEG patients, EEG source localization had a significantly higher concordance (92% versus 65%), sensitivity (57% versus 35%) and positive predictive value (60% versus 36%) compared with epileptologist localization. Our study demonstrates good concordance between ictal and interictal source imaging and intracranial EEG. Temporal lobe discharges have higher concordance rates than extratemporal discharges. Importantly, this study shows that source imaging has greater agreement with intracranial EEG than visual review alone, supporting its role in surgical planning.

## Teaser

EEG source imaging of 87 patients with focal epilepsy demonstrated overall concordance of ictal and interictal discharges with intracranial EEG. Improved concordance was seen with temporal discharges and with ictal spiking compared to other waveforms. Source imaging demonstrated improved concordance compared to epileptologist review in a subgroup of high-density EEG.

## Graphical Abstract

**Graphical Abstract:**
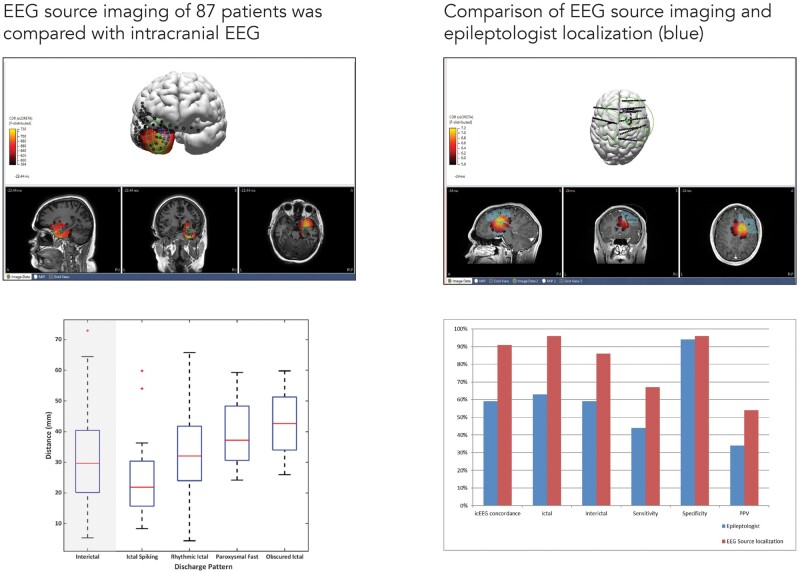

## Introduction

EEG source imaging (ESI) is an inexpensive and non-invasive diagnostic test for presurgical evaluation for medically refractory focal epilepsy. The challenge of scalp EEG source localization has been present for decades, as electroencephalographers have attempted to unravel the relationship between measured scalp EEG potentials and their cortical generators.^1^ Early source localization efforts assumed a single focus of activity using a uniform spherical distribution to model the brain, although neither assumption is physiologically accurate.^2–6^ Despite their simplicity, early dipole localization methods were able to localize interictal EEG activity to within the surgical resection site in small case series.^4^^,^^5^^,^^7^ (More sophisticated head models using the patient’s MRI and taking into account the different electrical conductivities of scalp, skull and brain tissues were developed, presumably producing more accurate localizations).^4^^,^^8^^,^^9^ Typical anatomical models include boundary element method (BEM), finite element method or the more simple local spherical head model with anatomic constraints, all of which have shown similar accuracy in localizing interictal spikes within surgical resection margins.^8^^,^^10^ Furthermore, the evolution of distributed current density source localization algorithms for solving the inverse problem has allowed for more anatomically consistent solutions than single point-source dipoles, although it is not clear whether these solutions are more accurate.^9^^,^^11^^,^^12^ A variety of more advanced algorithms has emerged in recent years, including minimum norm, low-resolution electromagnetic tomography (LORETA), sLORETA, eLORETA, local autoregressive average (LAURA), beamforming and MUSIC, each of which has potential advantages and disadvantages.^10^ A recent review article reported over 42 different statistical methods for EEG source localization.^13^ Although distributed solutions may be conceptually easier to interpret because of their visual appearance, to date, the literature has not shown that such models are more accurate representations of the true epileptogenic region. Several features, such as the spatial extent of the solution, can be adjusted with no clear standards or guidelines, which necessitates some caution in interpreting distributed current density solutions.

As techniques for ESI have advanced, its utility as a presurgical tool has become better appreciated. Brodbeck et al.^14^ evaluated the accuracy of ESI of interictal discharges in 152 surgical patients with good outcomes, using the surgical resection area as a reference standard. Their study demonstrated that ESI using high-density coverage (128–256 channels) of 55 patients yielded a sensitivity and specificity of 84 and 88%, respectively, which were higher than ictal single photon computerized emission tomography (SPECT), fluorodeoxyglucose postron emission tomography (FDG-PET) and structural magnetic resonance imaging (MRI). Koren et al.^15^ performed prospective ESI on ictal discharges in 28 presurgical patients with primarily temporal lobe epilepsy, demonstrating sensitivity 92.3% and specificity 60%, using surgical resection and seizure-free outcome as a reference. In a cohort of 87 patients, Sharma et al.^16^ utilized both dipole and LORETA solutions from low-density, 25-channel EEG to demonstrate agreement of interictal and ictal ESI with intracranial EEG (icEEG) and concordance with surgical resection between 51% and 62%. In this study, sublobar concordance was defined as an exact match between sublobar localizations from ESI and from icEEG and surgical resection. A recent meta-analysis of three studies found a sensitivity of 82% and specificity of 53% of ESI compared to surgical resection cavity in seizure-free patients.^17^

Despite prior studies of ESI, there remains uncertainty regarding optimal methods. Existing studies of the accuracy of ESI often report site-specific methods, parameters and choices of algorithms. High-density EEG arrays are important^14^^,^^18–23^ and are encouraged in the recent International Federation of Clinical Neurophysiology guidelines,^31^ but sometimes in clinical practice, only low-density EEG is practically feasible, particularly in prolonged monitoring.^11^^,^^16^^,^^24^ Ictal and interictal discharges can be mapped with ESI, but it is not clear which is more accurate and/or reliable, or whether particular seizure patterns produce more or less accurate results. A recent study saw no significant differences between ictal and interictal analyses using low-density EEG arrays,^16^ although a meta-analysis of several ESI and magnetic source imaging studies showed higher accuracy and sensitivity of ictal ESI compared to other modalities.^25^ Validation of ESI methods by comparing with surgical resection can be problematic, as surgical margins expand the putative seizure onset zone (SOZ) beyond truly pathological regions. Many patients do not ultimately undergo resection and may be treated by neuromodulation instead, and these patients would be overlooked in many prior validation studies. Finally, ESI may have a useful role in identifying patients who are poor candidates for surgery due to multifocality or involvement of eloquent brain areas, but there are little data in the ESI literature on patients who do not undergo resective surgery. We sought to answer several questions in a large retrospective cohort of patients with medically refractory focal epilepsy:

- What is the sublobar concordance between ESI and icEEG and what clinical factors are associated with concordance?
- For patients who do undergo surgical resection, are any features of ESI predictive of surgical outcome?
- Does ESI add localization value to what an experienced epileptologist may provide using scalp EEG and video alone?

## Materials and methods

We retrospectively evaluated all patients who had undergone icEEG, either stereotactic EEG (sEEG) or subdural grids, for medically refractory focal epilepsy in our institution between 1 September 2015 and 30 November 2018. Patients who met the following inclusion criteria were included: (i) medically refractory focal epilepsy; (ii) had undergone prolonged scalp EEG monitoring in our epilepsy monitoring unit (EMU); (iii) had MRI brain images available in our institutional archive; and (iv) had typical seizures captured on both EMU EEG and icEEG. Patients were excluded from the study if no seizures were captured in EMU or icEEG, or habitual seizures were not captured in EMU or during icEEG.

### Clinical data acquisition

Patients with medically refractory focal epilepsy underwent prolonged video-scalp EEG monitoring with either 76-channel high density (10–10 system) or 32-channel (10–20 system with subtemporal electrodes) electrode arrays in a dedicated adult or paediatric EMU. Six millimetre tin electrodes (LifeSync Neuro, Rochester, MN, USA) were affixed to the scalp with collodion paste, and EEG was recorded on an XLTEK acquisition system (Natus Inc., Pleasanton, CA, USA) at 250 or 500 Hz sampling rate using a reference near the vertex and an independent ground channel. As the majority of source imaging was done retrospectively after patients had completed monitoring, electrode locations were not digitized, but rather label matching was used. Idealized locations based on the 10–10 or 10–20 systems were utilized, with positions being calculated relative to individualized anatomic landmarks based on the patient’s MRI skin surface. EEG review was performed by dedicated technologists and board-certified physicians to identify seizures and interictal epileptiform discharges of interest.

Clinical information was obtained by retrospective chart review. Patient demographic information, epilepsy characteristics including prior surgeries, the presence of MRI lesion, as well as pathology and post-operative seizure freedom for patients who underwent a destructive procedure were recorded. Pathology results were grouped as ‘lesional’ referring to a pathology associated with epilepsy (e.g. hippocampal sclerosis, focal cortical dysplasia, etc.) or gliosis/normal cortex.

### EEG source imaging

Source imaging was performed using Curry 8.0 software (Neuroscan Compumedics, Charlotte, NC). ESI analysis was performed by two of our authors (B.C.C. and B.H.B.). Both authors performed ESI on a series of seven patients to assess inter-rater consistency, and agreement was measured using Cohen’s Kappa. All ESI analyses apply a common average reference to EEG channels during calculation of ESI solutions, but other montages were available during analysis to aid selection of analysis epochs.

Twenty patients had prospective ESI performed as part of their original pre-surgical assessment; the rest were evaluated retrospectively with the operator blinded to icEEG features and results.

ESI was performed using both moving dipole source localization and sLORETA current density.^26^ MUSIC dipole analysis was used for analysis of EEG epochs.^27^ The sLORETA displays threshold was set to 80% of the maximum, which was empirically chosen to provide a consistent display of results. Noise estimation was performed automatically by the analysis software, which uses the standard deviation of the bottom 50th percentile of sample values.

### ESI of interictal discharges

Interictal spikes were grouped based on common spike morphology and averaged to improve signal-to-noise ratio (SNR); a minimum of 10 spikes were averaged per analysis. Averaged interictal spikes were identified as propagating spikes using sequential 3D voltage maps and principal component analysis (PCA)^28^ and were analysed at 50% of peak amplitude on the ascending portion; non-propagating spikes were analysed at peak to maximize SNR.

### ESI of ictal discharges

Seizure discharges were analyzed using the earliest apparent electrographic change and were analyzed until activity either spread to adjacent channels or evolved significantly in frequency or morphology. To exclude EEG artefact, the analysis epoch was limited if possible, or independent component analysis (ICA) was used if artefact contaminated the ictal EEG onset. Band filters and ICA were used to restrict ictal signals closest to the frequency identified at ictal onset. Electrode channels containing persistent artefact that was not removed were excluded. Signal peaks of the filtered epoch were then averaged and ESI was performed. Our technique was similar to that described previously,^22^^,^^29^^,^^30^ with the exception that fast Fourier transformation was not performed on shorter segments, but rather the epoch of initial ictal onset was determined using visual inspection of the raw EEG signal in bipolar montage. Ictal EEG patterns were categorized as ictal spikes, rhythmic activity, paroxysmal fast activity or obscured (if cerebral activity was visually obscured by artefact).^18^

**Figure 1:**
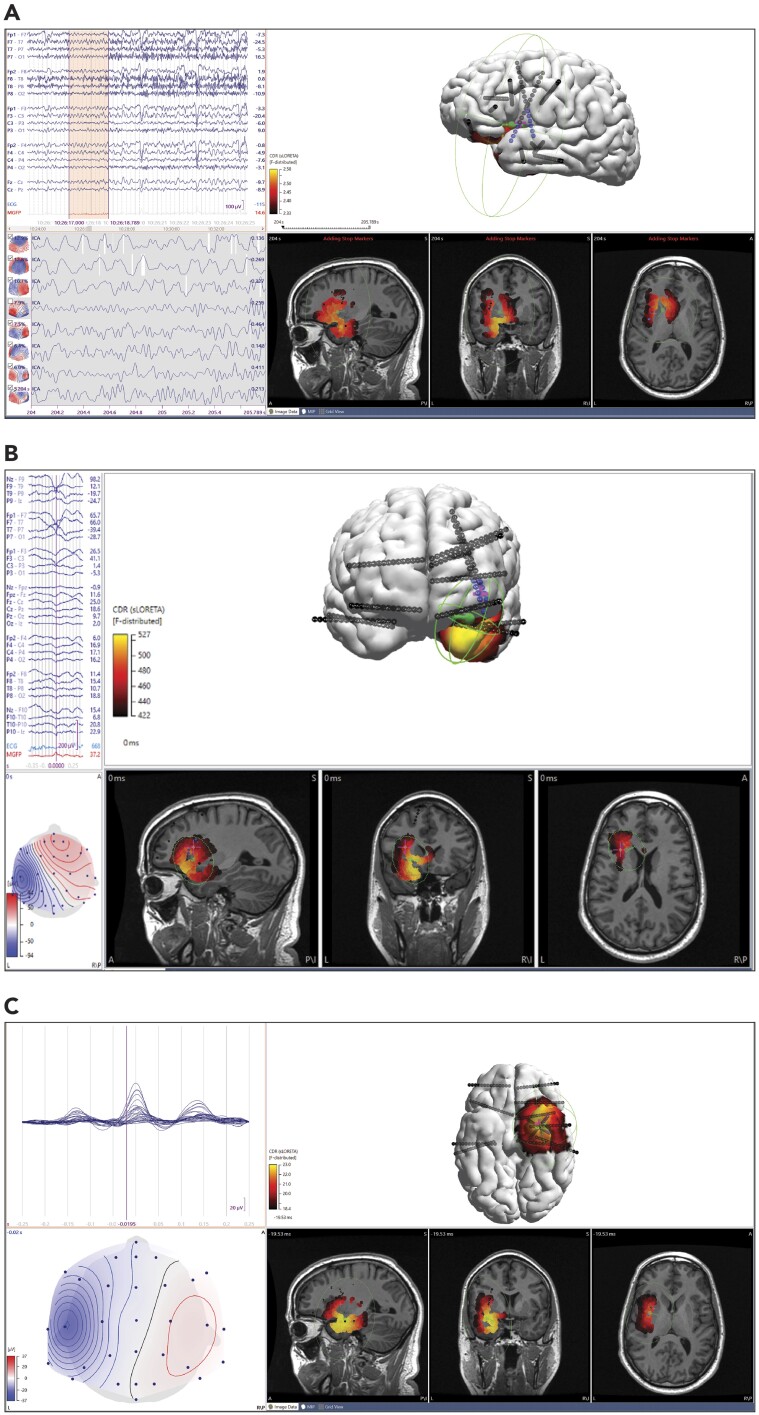
**ESI results of a non-lesional patient with standard density EEG. ESI dipole (green), intracranial electrodes (black, blue for seizure onset zone) and centroid of seizure onset zone (magenta cross).** Sublobar localization of intracranial EEG was left insula. Resection was not performed due to eloquent cortex, but patient has been seizure-free for 2 years with chronic subthreshold stimulation in left insula. (**A**) initial 2 s of ictal onset. sLORETA sublobar localization was left insula, left mesial frontal. Dipole-centroid distance (using fixed MUSIC) was 18.1 mm and to nearest SOZ electrode was 10.9. MSL-centroid distance was 19.5 mm and to nearest SOZ electrode was 12.2 mm. SNR 1.38. (**B)** Average of 14 interictal broad sharp waves, non-propagating. sLORETA sublobar localization was left insula and left temporopolar. Dipole centroid distance was 15.3 mm and to nearest SOZ electrode was 15.2 mm. MSL-centroid distance was 28.1 mm and to nearest SOZ electrode was 21.4 mm. SNR 1.90. (**C**) Average of 32 sharp waves, propagating. sLORETA sublobar localization was left mesial temporal, anterior, temporal and insula. Dipole-centroid distance was 23.5 mm and to nearest SOZ electrode was 16.6 mm. MSL-centroid distance was 23.8 mm and to nearest SOZ electrode was 19.5 mm. SNR1.8.

Source localizations were calculated using BEM head model created from the patient’s T_1_-weighted magnetization-prepared rapid gradient echo MRI sequence. Fifteen patients had 1.5 T MRI due to the presence of vagal nerve stimulator; all others had 3 T MRI, and three patients also had 7 T MRI. Brainstem and cerebellum were masked out of the head model if needed to constrain ESI solutions to grey matter regions within the supratentorial brain (Fig. 1). sLORETA ESI results were localized to the following 26 left or right sublobar brain regions: frontopolar, superior frontal, mesial frontal, inferior frontal, superior parietal, mesial parietal, inferior parietal, mesial occipital, lateral occipital, mesial temporal, lateral temporal temporopolar and insular (adapted from Schneider et al^31^). All sublobar regions that contained high-intensity (>80% threshold) sLORETA signal were included. Sublobar region categorizations were assigned with the reviewer blinded to icEEG results. Sublobar region localization was not assigned for dipole solutions, as these point source approximations are understood to be slightly offset from the cortex generating the discharges.^32^

### Intracranial EEG

The SOZ was determined by reviewing clinical icEEG reports. Intracranial electrodes that were active initially at seizure onset were included, and electrodes that later became active with propagation were excluded based on the clinical judgement of the reviewing epileptologist; this was confined to electrodes containing the first ictal change, similar to definitions of SOZ used in previous studies.^33–35^ While it might be possible for this initial change to represent seizure propagation if no electrodes sampled the actual seizure onset, the use of the centroid of SOZ contacts mitigates this potential limitation. Interictal discharges were not considered in determination of SOZ. All active SOZ icEEG electrodes were co-localized to patient’s MRI and BEM head model, and the sublobar localization of the icEEG SOZ was assigned, as was done with ESI. A sample including raw EEG, source localization and sEEG seizure onset is included in the Supplemental material (Supplementary Fig. S1).

### Concordance

Each sLORETA source localization was considered concordant if it overlapped icEEG in at least one sublobar region. Concordance for individual analyses was assigned in binary fashion (concordant or not concordant) with no consideration of partial concordance or proximity. A patient was categorized as minimally concordant if they had at least one concordant analysis, and completely concordant if all their analyses were concordant with icEEG. As most patients had more than one analysis, this provided a summary of patients’ overall concordances but did not distinguish between patients, for example, with one out of four analyses concordant or four out of five analyses concordant. As such, individual ESI analysis results were analysed separately as well.

If possible, the SOZ was categorized as temporal or extratemporal (insula was considered extratemporal). Patients whose SOZ involved both extratemporal and temporal structures were excluded from this subgroup analysis. The SOZ was classified as multifocal if two or more independent, non-adjacent foci of seizure onset were identified on icEEG. Each patient’s ESI was similarly classified as multifocal if results suggested two independent, non-adjacent foci of seizure onset.

### Dipole-centroid and sLORETA maximum-centroid comparison

In order to quantify the accuracy of ESI localization, the centroid of active icEEG SOZ electrodes was calculated and the distances were then measured between this point and both ESI dipole and the maximum point of sLORETA signal. Coordinates for icEEG electrodes were obtained from the patient’s post implant non-contrast head CT, which was then co-registered to and superimposed on the MRI brain and BEM head model using a normalized mutual information algorithm (Fig. 1). The centroid was calculated as the mean of all SOZ electrode coordinates with no differential weighting applied. Patients with multi-focal SOZ were excluded from dipole-centroid distance analysis. In addition, the distances were measured from dipoles and maximum sLORETA (MSL) to the nearest icEEG electrode in the SOZ, so as to allow more direct comparison with prior studies.^8^^,^^20^^,^^36^

### Surgical outcomes

A subgroup analysis was performed to identify characteristics associated with excellent post-surgical outcomes. Patients who had undergone surgical resection and had 1-year follow-up were analysed. Surgical outcomes at 1 year were grouped into Engel classification 1 (1A: Completely seizure-free since surgery, 1B: Non disabling simple partial seizures only since surgery, 1C: Some disabling seizures after surgery, but free of disabling seizures for at least 2 years, 1D: Generalized convulsions with antiepileptic drug withdrawal only) or greater than 1 (2: rare disabling seizures, 3: worthwhile improvement or 4: no worthwhile improvement).^37^ Patients treated with neuromodulation were not included in surgical outcomes. Patients who had focal laser ablation or a disconnection surgery were included.

### Epileptologist localization

Using a subset of 26 patients with high-density EEG, we assessed the accuracy and precision of ESI localization compared with the localizing information obtained during scalp EEG review by an experienced epileptologist. There were a total of 29 patients with high-density EEG in the study; however, three patients were initially miscategorized as standard density EEG at the time of the epileptologist review. Three experienced epileptologists (L.W.-K., B.N.L. and T.D.L.) reviewed the patients’ interictal and ictal video-EEG and assigned sublobar localizations. Reviewers were blinded to icEEG results and were excluded from reviewing patients with whose care they had been involved. Reviewers were provided with a head map showing electrode locations and a list of possible sublobar locations and anatomic boundaries. EEG was reviewed using a longitudinal bipolar high-density montage; however, epileptologists were free to review in other montages at their discretion. They were asked to localize ictal and interictal discharges and to assess whether SOZ was multifocal or unifocal. Reviewers were allowed to review concurrent video and consider semiology. They were also asked to localize seizure onset to a maximum of 3 of the 13 sublobar regions in either hemisphere (26 in total), with the option of ‘non-localizing’ if they felt that neither ictal nor interictal discharges were clearly localizing. Review was limited to patients with high-density EEG, as this was thought to give the epileptologists the best chance of being able to localize on a sublobar level. All three epileptologists were asked to review the same initial five patients to assess inter-rater reliability, and Fleiss Kappa was calculated to determine agreement (given more than two reviewers). The remaining 21 patients were then divided randomly among the reviewers.

### Statistics

All statistics were calculated using JMP 14.1.0. Concordance was compared between subgroups using a two-tailed Fisher’s exact test. Concordance rates between dipole ESI and sLORETA ESI was compared using McNemar’s test. Dipole-centroid and MSL-centroid distances were compared using Wilcoxon rank sum test, as data were not normally distributed. Comparisons were made among patients and between individual source localization analyses performed, regardless of patient. Statistics were calculated with and without multifocal SOZ patients excluded. Agreement between ESI and icEEG for detection of multifocal SOZ was assessed using kappa coefficients. In comparing epileptologist localizations with ESI, sensitivity, specificity, positive predictive value and negative predictive value were calculated for each, using icEEG SOZ localization as true positive. Sublobar regions for each patient were scored as true positives if both icEEG and scalp EEG (ESI or epileptologist visual review) identified the region as SOZ. Conversely, sublobar regions that icEEG and scalp EEG (ESI or expert review) had both identified as non-SOZ were considered true negatives. Since the epileptologists were restricted to choosing no more than 6 (up to 3 in either hemisphere) of 26 possible sublobar regions, there would be a minimum of 14 true negatives for each patient. Given that sublobar localizations were limited to 6 regions out of 26 possible sublobar localizations, the minimum true negative count (14) was subtracted from the true negatives to avoid biasing the results due to the experiment design (see Supplementary Data Analysis C). Sensitivity and specificity rates for identifying SOZ were calculated and were compared using McNemar’s test.^38^ Agreement between epileptologist review and ESI on detection of multifocal SOZ was compared using kappa coefficients. Outcomes of surgical resection in the subset of patients who had undergone surgery and had 1-year follow-up were compared between subgroups using odds ratios and Fisher’s exact test.

### Data availability

Deidentified data for this study are available in excerpted form upon reasonable request to the corresponding author.

## Results

### Patient results

**Figure 2:**
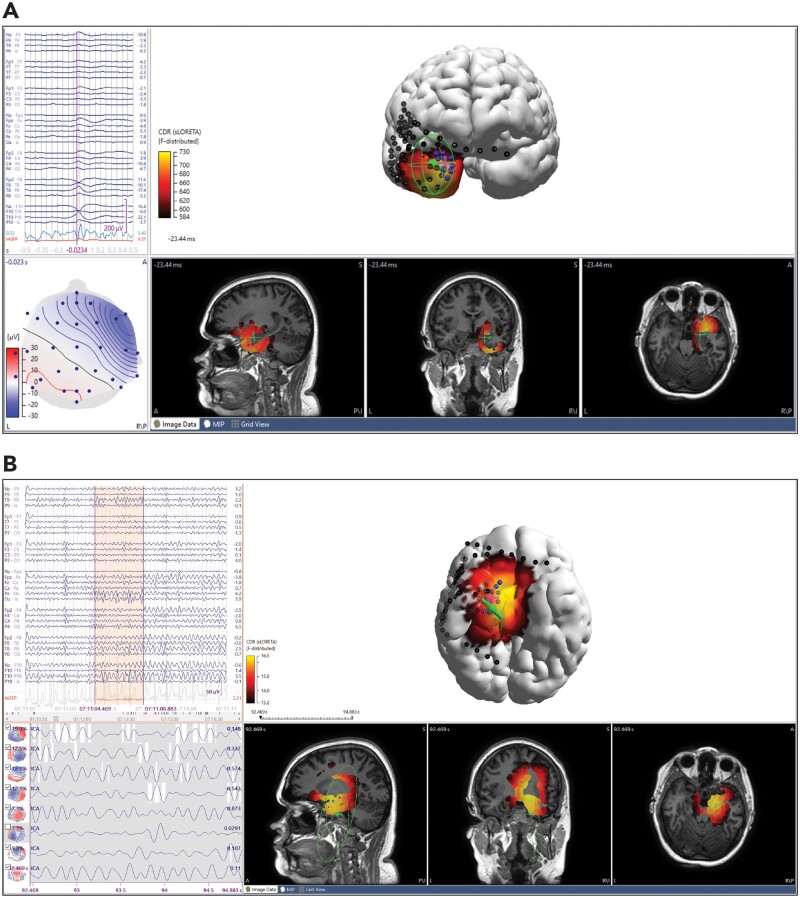
**ESI results of ictal and interictal standard density EEG in a non-lesional patient with subdural grids and strips. ESI dipole (green), intracranial electrodes (black, blue for seizure onset zone) and centroid of seizure onset zone (magenta cross). Sublobar localization of icEEG was right mesial temporal, right temporopolar. The patient was Engel class 1A at 1-year follow-up after anterior temporal lobectomy.** (**A**) Average of 14 interictal sharps, propagating. sLORETA sublobar localization was right mesial temporal, right temporopolar. Dipole-centroid distance was 8.3 mm and to nearest SOZ electrode was 6.7 mm. MSL-centroid distance was 12.4 mm and to nearest SOZ electrode was 6.5 mm. SNR 4.5. (**B**) Initial 2 s of ictal onset. sLORETA sublobar localization was R mesial temporal, insula, inferior frontal. Dipole-centroid distance (using fixed MUSIC dipole) was 27.7 mm and to nearest SOZ electrode was 16.8. MSL-centroid distance was 29.4 mm and to nearest SOZ electrode was 19.7 mm. SNR 2.9.

**Figure 3:**
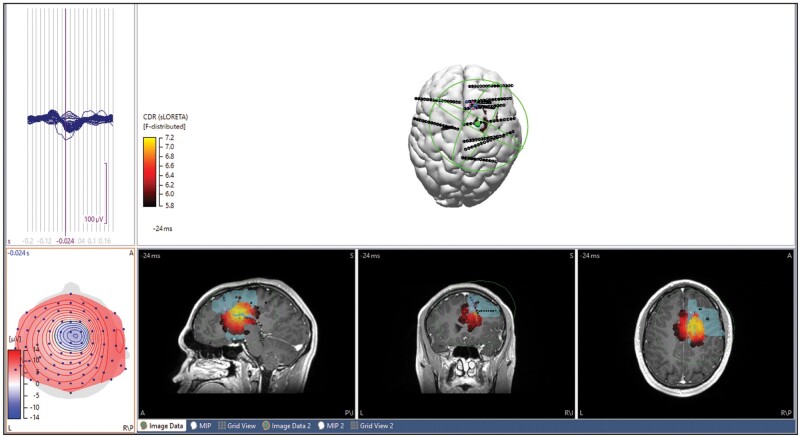
**ESI results of high-density EEG in a non-lesional patient with sEEG. ESI dipole (green), intracranial electrodes (black, blue for seizure onset zone) and centroid of seizure onset zone (magenta cross).** Sublobar localization of icEEG was right mesial temporal, right temporopolar. Sublobar localization of epileptologist was right mesial frontal and superior frontal (blue shading). The patient did not undergo resection due to overlap with eloquent cortex. sLORETA localization was right mesial frontal. Dipole-centroid distance (using fixed MUSIC) was 15.6 mm and to nearest SOZ electrode was 13.0 mm. MSL-centroid distance was 17.2 mm and to nearest SOZ electrode was 15.2 mm. SNR 1.4.

**Figure 4:**
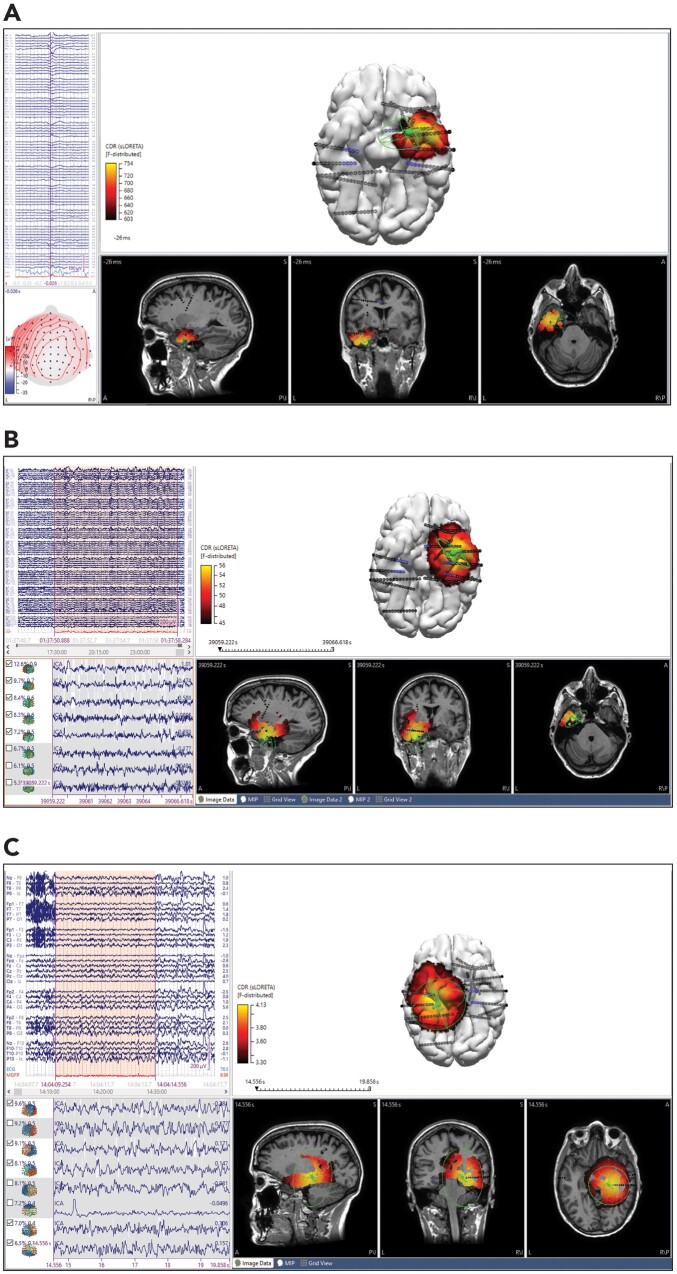
**ESI results of ictal and interictal high-density EEG in a non-lesional patient with sEEG. ESI dipole (green), intracranial electrodes (black, blue for seizure onset zone) and centroid of seizure onset zone (magenta cross). Sublobar localization of icEEG was independent right and left mesial temporal SOZ (multifocal). The patient enrolled in an investigational brain stimulation trial.** (**A**) Average of 14 propagating sharp waves. sLORETA localization was left mesial temporal, temporal polar. SNR 4.3. (**B**) Initial 8 s of seizure onset. sLORETA sublobar localization was left mesial temporal, temporopolar. SNR 1.82. (**C**) Initial 5 s of ictal onset. sLORETA localization was right mesial temporal. SNR 2.4.

**Figure 5:**
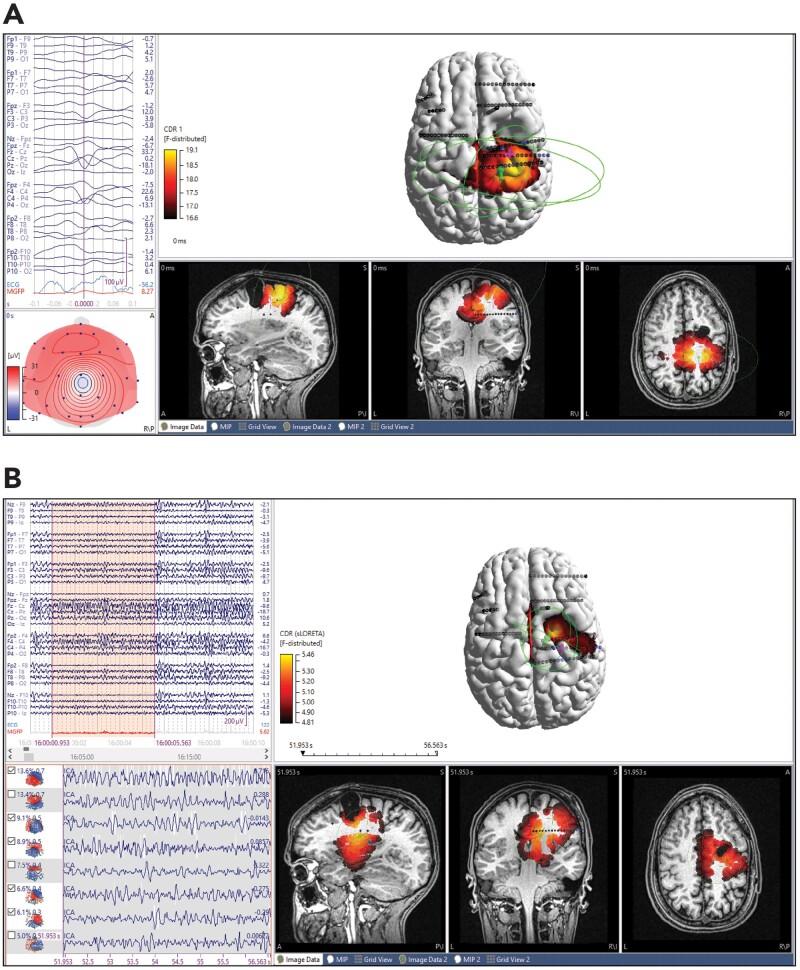
**ESI results of ictal and interictal standard EEG in a patient with prior right frontal resection and subsequent sEEG. ESI dipole (green), intracranial electrodes (black, blue for seizure onset zone) and centroid of seizure onset zone (magenta cross). Sublobar localization of icEEG was R frontal superior, frontal mesial. Patient underwent additional resection and was Engel class 3A at 1-year follow-up.** (**A**) Average 10 interictal sharp waves, non-propagating. sLORETA localization was right superior frontal and mesial frontal. Dipole-centroid distance was 18.9 mm and to nearest SOZ electrode was 6.3 mm. MSL-centroid distance was 16.9 mm and to nearest SOZ electrode was 5.2 mm. SNR 2.2. (**B**) Initial 5 s of seizure onset. sLORETA localization was right mesial frontal and inferior frontal. Dipole-centroid distance was 29.1 mm and to nearest SOZ electrode was 13.0 mm. MSL-centroid distance was 16.1 mm and to nearest SOZ electrode was 8.2 mm. SNR 3.4.

Ninety-three patients were identified who had undergone intracranial monitoring during the study period. Six of them were excluded: three had SOZ not captured on icEEG, one had additional seizure types on icEEG not captured during scalp monitoring and two patients had scalp EEG records that were not retrievable for review. There were 87 remaining patients with a total of 282 ESI analyses (155 ictal, 127 interictal) and median 3 ESI analyses per patient (range 1–7). Seven patients had only one analysis total, five patients had only interictal analyses and eight patients had only ictal analyses (see Figs 1–5 for sample results). Demographic information and baseline epilepsy characteristics are listed in Table 1. Inter-rater agreement between authors (B.C.C. and B.H.B.) on ESI sublobar localization was calculated in seven patients (12 total analyses) and determined to be substantial with Kappa 0.636 (see Supplemental Data Analysis A).

**Table 1:** Patient demographics and clinical characteristics of 87 patients

Characteristics	*n* (%)
Age (years) at time of EMU	
Median	25
Range	3–66
Gender	
Male	46 (53)
Female	41 (47)
Prior surgery	19 (22)
MRI strength	
1.5T	15 (17)
3 T	69 (79)
7 T	3 (3)
MRI	
Lesional	57 (66)
Non-lesional	30 (34)
SOZ location	
Temporal	43 (60)
Extratemporal	28 (39)
SOZ focality	
Multifocal	22 (25)
Unifocal	65 (75)
Scalp EEG density	
HD EEG (76 channel)	29 (33)
Standard EEG (32 channel)	58 (67)
ESI analyses	
Median total analyses per patient (range)	3 (1–7)
Ictal ESI	82 (94)
Median no. ictal analyses per patient (range)	2 (1–5)
Interictal ESI	20 (92)
Median no. interictal analyses per patient (range)	1 (1–6)
Both	74 (85)
Median no. total analyses per patient (range)	3 (1–7)
icEEG type	
Stereo EEG	57 (66)
Subdural grids	30 (35)
Intervention	
Surgical resection or LITT	57 (66)
Other	30 (37)
1-year surgical outcome (*n* = 52)	
Engel 1(a–d)	38 (73)
Engel 2–4	14 (27)
Pathology (*n* = 51)	
Lesional	33 (65)
Gliosis or normal cortex	18 (35)

Of the 87 patients studied, 83 (95%) had minimum concordance and 64 (74%) had complete concordance between sLORETA and icEEG (Table 2). There was no significant difference in minimal concordance between temporal and extratemporal SOZ; however, there was a significantly higher rate of complete concordance in patients with temporal SOZ compared to extratemporal (Table 2, 89 and 60%, respectively, *P* = 0.014). A Kappa coefficient of 0.49 demonstrated moderate agreement (0.4–0.6) between ESI and icEEG in detecting a multifocal SOZ (see Fig 4 for multifocal example). There was no significant difference found in concordance rates between patients based on EEG array density, icEEG type, lesional MRI, prior surgery or subsequent pathology in post-resection patients.

**Table 2:** Patient concordance rates

Subgroups	Minimum concordance, *n* (%)	Complete concordance, *n* (%)
All patients (*n* = 87)	83 (95)	64 (74)
Engel Class 1 at 1 year after surgery (*n* = 52)	51 (98)	37 (71)
Temporal (*n* = 28)^†^	28 (100)	25 (89)^*^
Extratemporal (*n* = 43)^†^	40 (93)	26 (60)^*^
sEEG (*n* = 60)	58 (97)	41 (68)
Subdural grids (*n* = 27)	25 (93)	22 (81)
High-density EEG (*n* = 28)	27 (96)	20 (71)
Standard EEG (*n* = 59)	56 (95)	43 (73)
ESI analyses		
Ictal (*n* = 82)	79 (96)	59 (72)
Interictal (*n* = 80)	76 (95)	57 (71)
Both (*n* = 74)	71 (96)	52 (70)
Prospective ESI (*n* = 20)	20 (100)	16 (80)
Retrospective ESI (*n* = 67)	63 (94)	47 (70)
MRI		
Lesional (*n* = 58)	55 (96)	39 (68)
Non-lesional (*n* = 29)	28 (93)	24 (80)
Prior surgery (*n* = 19)	19 (100)	15 (79)
No prior surgery (*n* = 68)	64 (94)	48 (71)
Pathology (*n* = 51)		
Lesional (*n* = 33)	32 (97)	24 (73)
Gliosis/normal cortex (*n* = 18)	17 (94)	13 (72)

Concordance rates between ESI and icEEG SOZ based on minimum concordance (at least one analysis concordant) and complete concordance (all analyses concordant). Results shown exclude multifocal SOZ (for data excluding multifocal SOZ, see Supplementary Table S1). Statistically significant results are identified by asterisks.

* Significance at *P* < 0.05.

^†^ Excluding 16 patients with temporal and extratemporal SOZ.

### Individual ESI concordance

There were 155 ictal and 127 interictal analyses performed (Table 3), with no significant difference in concordance rates observed between these two groups. When ictal discharge types were compared, there was a non-significant trend towards increased concordance among ictal spikes compared to all other discharge types collectively. Significantly greater concordance was found in analyses performed with temporal SOZ compared to extratemporal SOZ analyses.

**Table 3:** Individual ESI analyses excluding multifocal SOZ

Subgroups	Sublobar concordance, *n* (%)	Median dipole-centroid (mm) (IQR)	Median dipole–nearest SOZ electrode (mm) (IQR)	Median MSL-centroid (mm) (IQR)	Median MSL–nearest SOZ electrode (mm) (IQR)
All analyses (*n* = 221)	189 (86)	30.0 (21.3–40.2)	22.8 (14.0–32.1)	32.5 (21.8–43.0)	21.7 (13.3–33.4)
Ictal (*n* = 155)	131 (85)	30.6 (21.9–40.2)	20.3 (12.1–30.6)	31.8 (22.1–42.7)	19.3 (12.3–31.9)
Interictal (*n* = 127)	104 (82)	30.0 (20.2–40.7)	23.6 (15.4–36.3)	30.9 (20.5–43.2)	22.5 (14.8–36.1)
Ictal spike (*n* = 25)	24 (96)	21.9 (15.6–30.8)^**^	16.1 (11.3–21.8)^**^	26.5 (15.1–32.6)^**^	16.0 (8.6–21.4)^**^
Other (*n* = 82):	78 (82)	33.8 (24.6–43.0)	25.7 (17.3–37.9)	34.6 (22.6–44.5)	25.1 (17.0–37.4)
Rhythmic (*n* = 86)	81 (94)	32.1 (23.8–42.0)^a^^,^^*^	24.4 (16.1–35.5)^a^^,^^**^	32.5 (22.3–44.2)^a^^,^^*^	22.8 (15.9–5.7)^a^^,^^**^
PFA (*n* = 4)	3 (75)	37.2 (27.4–53.8)	33.2 (14.7–46.9)	35.2 (27.6–54.8)	32.3 (15.8–48.4)
Obscured (*n* = 10)	7 (70)	42.7 (32.5–51.5)	41.3 (25.4–45.7)	42.7 (35.7–50.5)	39.7 (25.4–43.6)
Temporal (*n* = 78)^b^	74 (95)^*^	28.8 (20.6–36.5)	19.5 (11.4–26.1)^**^	28.9 (21.9–38.2)	17.0 (11.3–26.3)^**^
Extratemporal (*n* = 121)^b^	95 (79)^*^	29.8 (20.7–42.7)	24.9 (15.2–36.3)^**^	32.6 (20.6–44.2)	24.1 (16.2–36.1)
sEEG (*n* = 136)	115 (85)	29.4 (20.1–41.0)	23.6 (14.5–32.7)	32.2 (20.5–42.7)	23.4 (14.0–35.2)
Subdural grids (*n* = 85)	74 (87)	30.6 (23.0–40.2)	21.7 (11.9–32.4)	30.8 (23.3–43.4)	20.4 (12.3–32.1)
High-density EEG (*n* = 68)	55 (81)	32.5 (22.2–42.8)	22.2 (13.7–31)	31.8 (19.8–42.0)	21.9 (12.3–35.9)
Standard EEG (*n* = 153)	134 (88)	29.8 (21.1–38.6)	24.6 (13.9–35.6)	31.1 (22.1–43.6)	21.7 (13.9–33.4)
Prospective (*n* = 43)	40 (93)	28.7 (17.4–33.8)^*^	20.8 (15.1–24.6)	28.7 (17.4–33.8)^*^	20.8 (15.1–24.6)
Retrospective (*n* = 178)	149 (84)	32.9 (22.1–43.7)^*^	22.7 (13.1–35.9)	32.9 (22.1–43.7)^*^	22.7 (13.1–35.9)
MRI lesional (*n* = 152)	121 (82)	31.5 (23.3–42.2)	23.5 (15.3–36)	32.7 (23.2–43.8)	22.7 (14.5–36.1)
MRI non-lesional (*n* = 69)	68 (92)	28.7 (18.1–37.3)	20.3 (13.0–29.5)	30.7 (19.7–41.4)	20.8 (12.2–29)
Prior surgery (*n* = 40)	38 (95)	27.6 (21.1–34.7)	20.9 (12.9–30.0)	30.2 (18.4–38.3)	20.2 (13.0–31.8)
No prior surgery (*n* = 181)	151 (83)	31.5 (21.5–41.1)	23.2 (14.2–34.3)	31.9 (22.1–43.5)	22.3 (14.0–35.2)
Pathology (*n* = 142)					
Lesional (*n* = 78)	70 (90)^*^	29.9 (23.3–38.1)	23.2 (14.5–31.3)	30.6 (24.2–37.4)	21.9 (15.5–32.5)
Gliosis/normal (*n* = 64)	48 (75)^*^	31.6 (21.8–42.4)	24.5 (17.2–37.8)	33.1 (20.8–42.5)	23.2 (16.1–36.9)

Sublobar concordance of individual ESI results with icEEG SOZ is shown along with subgroup analyses. Results shown are excluding 61 analyses of patients with multifocal SOZ (for sublobar concordance including multifocal patients, see Supplementary material). Ictal waveforms were categorized as ictal spikes, rhythmic activity (not spike), paroxysmal fast activity (PFA) or obscured EEG onset. Also shown are dipole-centroid and dipole–nearest SOZ electrode distances, excluding multifocal SOZ. Significant results are identified by asterisks.

a Significant only when compared to Obscured Onset.

b Excluding 22 analyses of patients with SOZ involving both temporal and extratemporal regions.

* Significant with *P* < 0.05.

** Significant with *P* < 0.01.

After excluding 112 analyses performed on patients with multifocal SOZ, the median dipole-centroid and MSL-centroid distances were 30.4 mm and 31.4 mm, respectively (170 analyses). A Wilcoxon rank sum test demonstrated no significant difference between these two measurements in any of the subgroups examined—no difference was seen between ictal and interictal analyses, between temporal and extratemporal SOZ, between sEEG and subdural grid icEEG groups or between high density and standard density scalp EEG (Table 3). The median distances from dipole and MSL to the nearest SOZ electrode were 22.8 and 21.7 mm, respectively. Unlike centroid distances, both median dipole-nearest SOZ electrode and MSL-nearest SOZ electrode distances were significantly closer for temporal discharges compared with extratemporal ones. In looking at ictal waveform morphology, there was found to be a significantly smaller dipole-centroid distance (21.9 mm) and MSL-centroid distance (26.5 mm) for seizures with a repetitive spiking pattern at onset compared to all other ictal morphologies (Table 3 and Fig. 2). In this subset, analyses of ictal rhythmic activity were found to have significantly shorter dipole and MSL distances only when compared to obscured onset.

### Surgical outcomes

Of the 52 patients who underwent surgical resection (*n* = 47) or laser ablation (*n* = 5) and had follow-up at 1-year, 51 (98%) patients had minimal concordance and 37 (73%) had complete concordance (Table 2). Thirty-three patients with resection (65%) had a lesional pathology and 18 patients (35%) had normal cortex or non-specific gliosis. For specific pathologies, see Supplementary material. Thirty-eight patients (73%) had Engel class 1 outcomes (Table 4). Outcomes for patients with longer than 1-year follow-up were noted but did not differ from the 1-year Engel classification. No significant differences were seen across subgroups, examining minimal concordance, complete concordance and SOZ location. In total, 71% of individual analyses (114 out of 160 analyses) were associated with Engel class 1 surgical outcomes. There were no significant differences found in outcomes based on overall ESI concordance or concordance when comparing ictal and interictal, or temporal and extratemporal analyses. This contrasts with other studies, which have shown improved outcomes associated with colocalization of ESI to resection cavity. There was also no difference in concordances between subdural grids and sEEG, or between high-density EEG and standard EEG. The dipole-centroid distances and MSL-centroid distances were significantly smaller for ESI of patients with Engel class 1 compared to Engel class 2 or worse post-surgical outcomes (Table 5).

**Table 4:** Outcomes of 52 patients who had surgical outcomes at 1 year available.

	Engel Class 1	Engel Classes 2–4
Total patients (*n* = 52)	38 (73)	14 (27)
Minimal concordance		
sLORETA (*n* = 51)	37 (73)	14 (27)
Complete concordance		
sLORETA (*n* = 37)	29 (78)	8 (22)
Temporal seizure onset (*n* = 19)	16 (84)	3 (16)
Extratemporal Seizure onset (*n* = 25)	19 (76)	6 (24)

Percentages are in relation to subgroup. No significant differences were found.

**Table 5:** 1-year surgical outcomes associated with individual ESI analyses: Association between individual ESI analyses using maximum sLORETA (MSL) and patient surgical outcome at 1 year

Analyses	Engel Class 1, %	Engel Class ≥2, %	*P*-value, OR (95%CI)
Total analyses	114	44	
Concordant (*n* = 133)	98 (75)	35 (25)	0.16, 2.03 (0.770–5.22)
Discordant (*n* = 27)	16 (59)	11 (41)	
Ictal			
Concordant (*n* = 77)	55 (71)	22 (29)	0.18, 2.47 (0.591–10.37)
Discordant (*n* = 12)	6 (50)	6 (50)	
Interictal			
Concordant (*n* = 54)	43 (80)	11 (20)	0.31, 1.93 (0.45–8.29)
Discordant (*n* = 15)	10 (67)	5 (33)	
Temporal			
Concordant (*n* = 53)	46 (87)	7 (13)	0.57, OR N/A
Discordant (*n* = 4)	4 (100)	0 (0)	
Extratemporal			
Concordant (*n* = 50)	34 (68)	16 (32)	0.21, 1.93 (0.62–6.01)
Discordant (*n* = 23)	12 (52)	11 (48)	
sEEG			
Concordant (*n* = 78)	59 (76)	19 (24)	0.18, 2.13 (0.690–6.43)
Discordant (*n* = 22)	13 (59)	9 (41)	
Subdural grids			
Concordant (*n* = 53)	39 (74)	14 (26)	0.609, 1.83 (0.140–17.84)
Discordant (*n* = 5)	3 (60)	2 (40)	
Dipole-centroid (mm)	29.9	34.8	0.015*
Dipole-nearest SOZ	24.4	22.0	0.89
MSL-centroid (mm)	29.9	36.7	0.0035*
MSL-nearest SOZ	23.1	22.1	0.32

Percentages are in relation to Engel classification, not subgroup. MSL-centroid distances are reported as median distance in mm and compared using Wilcoxon Rank Sum. Significant results are identified by asterisks.

There were 12 patients who had prospective ESI and subsequently went on to have surgical resection with 1-year follow-up. There were 10 patients with Engel class 1 outcomes. All 10 had at least minimal concordance, and seven patients had complete concordance.

### Epileptologist review compared to ESI

The results from the 26 patients with HD EEG comparing epileptologist localizations to those obtained with ESI are reported in Table 6 (see Fig. 3 for example). Inter-rater agreement among the three epileptologists on first five patients (ictal and interictal) was substantial, with Kappa 0.659 (see Supplementary Data Analysis B). There was a significantly higher concordance with icEEG overall for ESI compared to the epileptologists, and this remained significant for both ictal and interictal subgroups. Sensitivity and positive predictive value were both significantly higher with ESI, but specificity and negative predictive values were not significantly different. Kappa coefficients for detection of multifocal SOZ suggested slightly higher agreement between ESI and icEEG than epileptologist and icEEG, but this difference was not statistically significant.

**Table 6:** Comparison between epileptologist review and ESI concordance with icEEG

	Epileptologist review, *n* (%)	EEG source localization, *n* (%)
Overall concordance with icEEG (*n* = 52)	33 (65)	47 (92)^**^
Ictal concordance (*n* = 26)	17 (65)	25 (96)^**^
Interictal concordance (*n* = 26)	16 (64)	22 (88)^*^
Sensitivity	34.8 (26.1–44.4)	57.4 (47.5–66.9)^**^
Specificity	85.7 (82.4–88.7)	90.9 (87.9–93.3)
Positive predictive value	35.5 (28.3–43.4)	59.6 (51.5–67.3)^*^
Negative predictive value	85.4 (83.6–87.1)	90.1 (87.9–91.9)
Detection of multifocal seizure onset zone (Kappa)	0.31 (0.0058–0.61)	0.49 (0.22–0.76)

* Significant with *P* < 0.05.

** Significant with *P* < 0.001.

## Discussion

This study demonstrates high rates of concordance between ESI and icEEG with high-density and low-density scalp EEG. Nearly all patients studied had at least one ESI analysis concordant with SOZ on the sublobar level, and 74% of patients had ESI results fully concordant with icEEG results. The reason for the higher rate of complete concordance in patients with temporal SOZ compared to extratemporal is not clear, but could be related to the number of spikes analysed in each analysis or differences in spike morphology, or perhaps sampling bias due to the larger number of sublobar regions in the extratemporal category. Temporal lobe ESI is aided by the presence of subtemporal chain electrodes in our standard EEG montage, which have been shown to add additional localizing value in temporal lobe epilepsy.^39^^,^^40^ It has been postulated that accurate localization of extratemporal lobe seizures requires a broader area of coverage including infraorbital EEG leads and high-density EEG,^41^ and infraorbital electrodes are not typically included in our electrode arrays. Surprisingly, we did not see a statistically significant increase in concordance rates, either minimal or complete, in patients with high-density EEG, although there was a non-significant trend towards increased complete concordance. Prior studies suggest using 120 or more electrodes can improve the accuracy of ESI, and it is possible that if we had used additional electrodes in our HD arrays or had more HD patients, we might have found a significant difference. It is also possible that the lack of digitization of EEG locations in our retrospective HD-EEG patients may have prevented finding a significant difference. As our study was retrospective and utilized the EEG montage selection at the time of EMU admission, patient-specific factors may have contributed to lower concordance rates than expected in the high-density group, as has previously been observed.^42^ It is also possible that our more liberal criteria for concordance (including partial concordance) may have decreased our ability to detect subtle differences in concordance rates. Our results suggest ESI is more spatially accurate than expert visual review of EEG alone. The higher sensitivity and positive predictive value suggest that ESI is able to localize SOZ more accurately and suggests it can help guide invasive EEG electrode planning. Kappa coefficients demonstrated a moderate degree of agreement with icEEG in detecting patients with multifocal SOZs for both ESI and epileptologist review, suggesting ESI may contribute little additional to this determination.

In analysing individual ESI analyses, a similarly high rate of concordance was observed in both ictal and interictal discharges, suggesting that both have localizing value in ESI. Ictal analyses are often more technically challenging due to the potential presence of myogenic and ocular artefacts and the limited ability to improve SNRs by waveform averaging, but have the advantage of directly studying the SOZ rather than the irritative zone as is done with interictal discharges. SOZ location (temporal versus extratemporal) was the most consistently significant variable, likely for the reasons discussed above. A significantly higher rate of concordance for subdural grid icEEG compared to sEEG was observed initially, but this difference was not significant with multifocal patients excluded.

Previous studies assessing the accuracy of ESI have compared the ESI localization to the nearest icEEG SOZ electrode, or to the edge of a lesion or resection bed.^8^^,^^20^^,^^36^^,^^43^ However, these measures will always underestimate the true distance, as the ESL solution should represent the centre of pathological activity, and surgical resection usually involves excising a margin of healthy tissue around the pathological tissue generating seizures. The centroid of icEEG SOZ electrodes is a more accurate measure, but as no prior study has used this, these measurements are difficult to compare directly. To facilitate comparison, we also reported the distance to the nearest SOZ electrode, and our results were similar to prior reports. Birot et al. had a median distance of 15.6 mm between ESI and the nearest SOZ electrode, using BEM models. Megavand et al. reported a median distance of 17 mm to the nearest SOZ electrode. Both of these measurements are closer than ours, but it should be noted that these studies utilized much higher EEG densities (128 or 256 channels), which may account for their greater accuracy. Lantz et al. reported a median distance of 6 mm from ESI to lesion border using 61 scalp electrodes and 22 mm using 31 electrodes. Sohrabpour et al. similarly measured a median localization distance of 10 mm using 32 electrodes and 6 mm using 64 electrodes to a surgical resection border. In addition to the above differences, all of these studies used interictal discharges only and did not perform ictal ESI, perhaps in part due to the logistical difficulty of acquiring prolonged high-density EEG recordings. We believe that using the icEEG SOZ centroid as a comparator is more reliable measure and better represents true localization error.

We found no significant difference between dipole-centroid and MSL-centroid distances. Interestingly, shorter distances were seen with temporal discharges when measuring to the nearest SOZ electrode compared to extratemporal discharges, a finding not observed when measuring to the centroid. The reasons for this are not clear, but could be related to the size of SOZ, which would affect distances measured to nearest electrode but not those measured to the centroid. Also, among ictal EEG morphologies, ictal spikes had significantly closer dipole-centroid and MSL-centroid distances, suggesting this waveform has greater localizing value in ESI than other ictal patterns. The significantly smaller dipole-centroid and MSL-centroid distances in ESI of patients with Engel Class 1 surgical outcomes may suggest that close agreement between ESI and icEEG is a favourable indicator of better post-resection outcomes. However, many factors we could not control for, like the total surgical resection volume and overlap with eloquent cortex, also influence post-resection outcomes. Surgical outcomes were not significantly affected by the rate of ESI sublobar concordance with icEEG SOZ. While the significance of the lower dipole-centroid and MSL-centroid distances for Engel 1 outcomes did not appear as significantly better concordance rates in our data, prior studies have shown high concordance of ESI within surgically resected areas in patients with favourable post-surgical outcomes).^14^^,^^15^^,^^44^ It may be that the low number of patients with poor outcomes in our cohort limited our ability to detect a difference in concordance as well.

There were several limitations to this study. As a retrospective analysis of EMU EEG recordings, patients were not randomized to high-density EEG or standard density EEG, but rather arrays were chosen at the discretion of the supervising physician. This may explain why we found no difference between HD EEG and standard EEG (in contrast to prior studies^14^), as patients with extratemporal or poorly localized scalp EEG may have been more likely to have HD EEG during EMU monitoring. Retrospective review bias was minimized by blinding epileptologists and ESI analysts to icEEG results, but such bias is difficult to eliminate entirely. An additional limitation is the limited spatial sampling achievable with icEEG. sEEG electrodes sample a very small proportion of brain tissue and if not well placed may not precisely identify the origin of seizures. Clinical subdural grids also have limitations, as they sample the gyral crowns at centimetre spacing, and discharges could originate in deep sulci and propagate some distance before being recorded by a contact. By using the centroid of the SOZ electrodes as our gold standard, we believe we minimized the impact of these factors, but there is undoubtedly some residual inaccuracy in this calculation. The study was further limited by the necessity of comparing scalp EEG and icEEG recordings acquired at different times. Ideally, simultaneous scalp and icEEG would be recorded and compared directly to eliminate any variation in the onset or propagation of different discharges, but this was not feasible in a retrospective study and is precluded by the clinical requirements for icEEG. However, this study does parallel the clinical progression from scalp EEG to icEEG to resection, and these data support ESI as having a contributory role in presurgical planning. Finally, epileptologists do not typically approach scalp EEG review by localizing discharges to a sublobar level; we attempted to mitigate this by providing reviewers with a map of electrode placements in relation to a brain model; however, it is still possible that the forced compartmentalization of EEG localization into sublobar regions may have falsely lowered the accuracy of the epileptologists in comparison to common clinical practice. Nevertheless, the large difference between ESI and epileptologist results suggests that ESI does offer increased localization value.

There are many facets of ESI that remain to be optimized, and additional studies are needed. While it is common practice to obtain as many interictal spikes as possible for averaging, there is little data guiding what is an acceptable number of spikes to analyse, or how similar spikes should be morphologically in order to be grouped and averaged. There are also many variables in ictal ESI that have not been well studied, including the use of ICA or PCA to remove artefacts, the duration of ictal epochs to be analysed and the trade-offs between ictal SNR and propagation.

To conclude, our study demonstrates good concordance between ESI using prolonged EEG monitoring with both 76-channel high density and 32-channel standard EEG arrays with sub-temporal electrodes. Both ictal and interictal ESI were accurate and showed high rates of concordance with icEEG, suggesting ESI can contribute to surgical planning. Temporal lobe discharges had higher icEEG concordance rates than extratemporal discharges, although the factors contributing to this are not clear. Importantly, this study showed that ESI had greater agreement with invasive icEEG results than visual review by experienced epileptologists alone, supporting ESI as a valuable contributory technique in surgical planning.

## Supplementary material

Supplementary material is available at *Brain Communications* online.

## Funding

This project was completed without external funding sources.

## Competing interests

The authors report no competing interests.

## Supplementary Material

fcab278_Supplementary_Data

## Abbreviations

BEM =: boundary element method
eLORETA =: exact low resolution brain electromagnetic tomography
EMU =: epilepsy monitoring unit
ESI =: EEG source localization
FDG-PET =: fluorodeoxyglucose-positron emission tomography
FEM =: finite element method
HD =: high density
ICA =: independent component analysis
icEEG =: intracranial EEG
LAURA =: local autoregressive average
LORETA =: low-resolution electromagnetic tomography
MPRAGE =: magnetization-prepared rapid gradient echo
MSL =: maximum sLORETA
MUSIC =: multiple signal classification
PCA =: principal component analysis
PFA =: paroxysmal fast activity
sEEG =: stereotactic EEG
sLORETA =: standardized low resolution brain electromagnetic tomography
SNR =: signal-to-noise ratio
SOZ =: seizure onset zone
SPECT =: single-photon emission computerized tomography
VNS =: vagal nerve stimulator
