# Supplementary material for: EEG source imaging concordance with intracranial EEG and epileptologist review in focal epilepsy
Source: Brain Commun. 2021 Nov 19;3(4):fcab278. doi: 10.1093/braincomms/fcab278 (PMC8643498; doi:10.1093/braincomms/fcab278)
Supplement: fcab278_Supplementary_Data [file fcab278_supplementary_data.docx]

**Supplementary material**

**Figure S1:** Shown is original high density (76 channel) EEG showing earliest ictal onset at P9/P5 (A) and EEG filtered using individual component analysis to subtract artifacts (B). Source localization results are shown in E with ictal dipole (red), interictal dipole (green, EEG not shown), all sEEG electrodes (blue) active sEEG leads at seizure onset zone (green), centroid of active leads (magenta cross), ictal sLORETA (red/yellow scale), and epileptologist review localization (tinted blue). Ictal and interictal dipole-centroid distances were 26mm and 39.7mm, respectively. Sublobar localization was lateral temporal, lateral parietal on both ESI and icEEG. Epileptologist localization was lateral temporal and medial temporal. Source localization results are shown on T2 FLAIR MRI (C), with ictal dipole changed to yellow. Stereo EEG onset is shown in referential montage at LAT 4-8, LIP 2-3, LBP 3-4 (D).


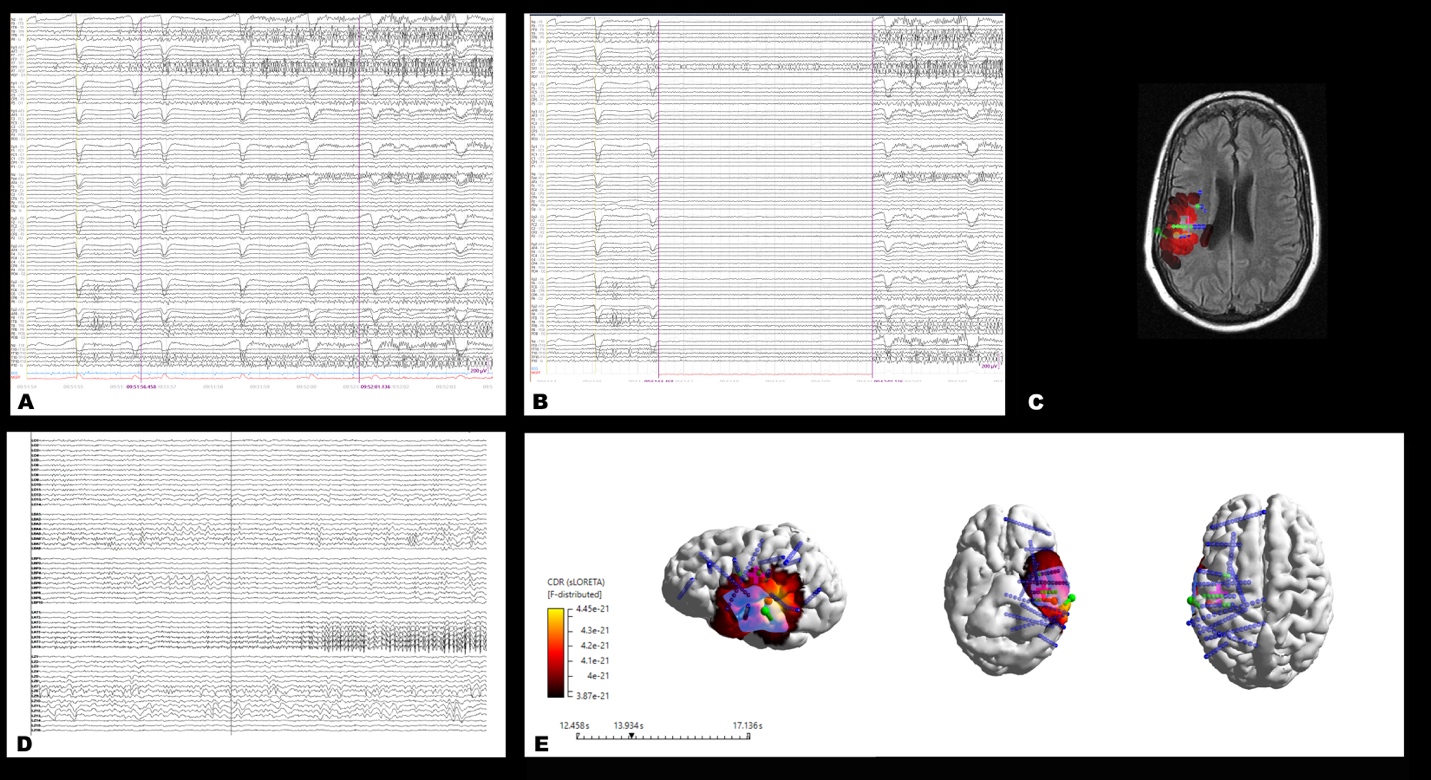


**Table S1**: Subgroup analysis excluding patients with multifocal seizure onset zone on icEEG

| Subgroups | Minimum Concordance,No.(%) | Complete Concordance, No. (%) |
| --- | --- | --- |
| All patients (n=87)  Excluding multifocal (n=65) | 83(95%)  62(95%) | 64(74%)  48(74%) |
| Temporal (n=21) †  Extratemporal (n=37) † | 21(100%)  35(95%) | 19 (90%)  24 (64%) |
| sEEG (n=43)  Subdural Grids (n=22) | 42(98%)  20(91%) | 31(72%)  17(77%) |
| High density EEG (n=23)  Standard EEG (n=42) | 22(95%)  40(95%) | 17(74%)  31(74%) |
| ESI analyses  Ictal  Interictal  Both | 59 (97%)  57 (97%)  52 (96%) | 43 (70%)  42 (71%)  37 (67%) |
| Prospective ESI(n=15)  Retrospective ESI(n=50) | 15 (100%)  48 (96%) | 12 (80%)  35 (70%) |
| MRI  Lesional (n=44)  Nonlesional(n=21)) | 42 (97%)  21 (95%) | 29 (67%)  18 (82%) |
| Prior surgery (n=13)  No prior surgery (n=52) | 13 (100%)  50 (96%) | 11 (85%)  36 (69%) |
| Pathology (n=51)  Lesional (n=26)  Gliosis/normal cortex (n=15) | 25 (96%)  14 (93%) | 19 (73%)  10 (67%) |

**Table S2**: subgroup analysis of ESI concordance including ESI of patients with multifocal SOZ. Significant results are identified by *.

| Subgroups | Sublobar Concordance, No. (%) | P value |
| --- | --- | --- |
| All analyses (n=282) | 235(83%) |  |
| Ictal (n=155)  Interictal (n=127) | 131(85%)  104(82%) |  |
| Ictal discharge type:  Ictal spike (n=38)  Other (n=117):  rhythmic activity (n=94)  paroxysmal fast activity (n=12)  obscured (n=11) | 34(89%)  78(82%)  81(94%)  9(75%)  7(64%) |  |
| Temporal (n=87) †  Extratemporal (n=133) † | 80(92%)*  99(74%)* | <0.05 |
| sEEG (180)  Subdural Grids (102) | 144(80%)*  91(89%)* | <0.05 |
| High density (76 lead) (94)  Standard (32 lead) (112) | 78(85%)  157(83%) |  |
| Prospective(n=48)  Retrospective(n=234) | 43 (90%)  192 (82%) |  |
| MRI lesional (n=196)  MRI nonlesional (n=86) | 154 (80%)  81 (89%) |  |
| Prior surgery (n=56)  No prior surgery (n=226) | 49 (88%)  186 (82%) |  |
| Pathology (n=170)  Lesional (n=94)  Gliosis/normal (n=76) | 79 (84%)  60 (79% |  |

†excluding 62 analyses with SOZ involving both temporal and extratemporal

**Table S3: Outcome data for patients with prospective ESI**

|  | Engel Class 1 | Engel Class 2-4 |
| --- | --- | --- |
| Total patients (n=12) | 10 | 2 |
| Minimal concordance (n=12) | 10 (83%) | 2 (17%) |
| Complete concordance (n=9) | 7 (78%) | 2 (22%) |
| Temporal Seizure onset (n=4)  Extratemporal Seizure onset (n=8) | 4 (100%)  6 (75%) | 0 (0%)  2 (25%) |
| MRI lesional (n=7)  MRI nonlesional (n=5) | 5 (71%)  5 (100%) | 2 (29%)  0 (0%) |
| Prior surgery (n=3)  No Prior surgery (n=9) | 2 (67%)  8 (89%) | 1 (33%)  1 (11%) |
| Pathology (n=10)  Lesional (n=5)  Gliosis/normal cortex (n=5) | 4 (80%)  4 (80%) | 1 (20%)  1 (20%) |

**Table S4: Pathology results**

| Pathology (n=51) | |
| --- | --- |
| Hippocampal sclerosis | 9 (18%) |
| FCD 1 (a-c unspecified) | 1 (2%) |
| FCD 1c | 1 (2%) |
| FCD 2a | 5 (10%) |
| FCD 2b | 5 (10%) |
| Cavernoma | 1 (2%) |
| DNET | 1 (2%) |
| PLNTY | 1 (2%) |
| Polymicrogyria | 1 (2%) |
| Tuber | 1 (2%) |
| Cystic encephalomalacia | 1 (2%) |
| Gliosis | 21 (41%) |
| Normal cortex | 3 (6%) |

**Supplementary data analysis:**

1. ESI source localization agreement between authors (BC and BB) on 12 analyses

(from 7 patients). Calculated using Cohan’s Kappa:

Kappa 0.636, SE 0.079, lower 0.480, upper 0.791

1. Interrater agreement on first 5 patients (ictal and interictal) analyses by 3 epilepsy reviewers. Calculated using Fleiss Kappa:

Kappa 0.659, SE 0.183, z 3.61, p value 0.00031

1. Adjustment for specificity calculation: total sublobar localizations were 26 (13 on either side) and sublobar localizations were limited to 6 regions. In a scenario of maximum error in either ESI or epileptologist localization, there would be 0 true positives, 6 false positives and 6 false negatives, resulting in a minimum of 14 (26-12) true positives. This was not thought to be clinically meaningful and so 14 was subtracted from true negatives prior to specificity calculations to adjust for this limitation.
